# Effects of Oral Vitamin B1 and Mecobalamin on Dry Eye Disease

**DOI:** 10.1155/2020/9539674

**Published:** 2020-09-09

**Authors:** Xiaotong Ren, Yilin Chou, Xiaodan Jiang, Ran Hao, Yuexin Wang, Yanyan Chen, Xuemin Li

**Affiliations:** ^1^Department of Ophthalmology, Peking University Third Hospital, Beijing, China; ^2^Department of Ophthalmology, Daqing Oilfield General Hospital, Daqing, Heilongjiang, China

## Abstract

**Purpose:**

To assess the effects of oral vitamin B1 and mecobalamin on dry eye disease (DED) and patient satisfaction with treatment.

**Methods:**

In this randomized controlled study, DED patients were divided into 4 groups based on treatment: group 1, only artificial tears; group 2, corticosteroid eye drops and artificial tears; group 3, oral vitamin B1, mecobalamin, and artificial tears; and group 4, oral vitamin B1, mecobalamin, corticosteroid eye drops, and artificial tears. DED symptoms, signs, and patient satisfaction with treatment were assessed at baseline and at 1 and 2 months after treatment.

**Results:**

In total, 152 eyes from 76 patients (age, 55.25 ± 18.16 years) were included. In group 3, there were significant differences in dryness, foreign body sensation, burning, and tear film breakup time first (TBUTF) between 1 and 2 months after treatment and in satisfaction scores before and after treatment (*P* < 0.05). In group 3, there were also significant differences in dryness, foreign body sensation, photophobia, and TBUTA and between baseline and 2 months after treatment (*P* < 0.05). There was a significant difference in foreign body sensation between 1 and 2 months after treatment in groups 3 and 4 (*P* < 0.05). Furthermore, we also find obvious improvement in corneal nerve fiber density (CNFD) between baseline and 1 and 2 months after treatment in groups 3 and 4 (*P* < 0.05).

**Conclusions:**

Oral vitamin B1 and mecobalamin can relieve some dry eye symptoms such as dryness, pain, and photophobia and improve DED signs and patient satisfaction. Thus, vitamin B1 and mecobalamin are potential treatment options for patients with DED.

## 1. Introduction

Dry eye is a multifactorial disease of the ocular surface characterized by loss of tear film homeostasis and ocular symptoms. Instability of the tear film, inflammation and injury of the ocular surface, and neurosensory abnormalities all play important roles in dry eye [1]. Pain related to dry eye disease (DED) is transmitted through the peripheral axons of trigeminal ganglion (TG) neurons innervating the cornea and conjunctiva [2]. The cornea is one of the densest nervous tissues [2] and is responsible for various sensations in the eye, such as pain, touch, and temperature. The trigeminal nerve also plays an essential role in the corneal reflex and tear production [3]. DED can cause damage to corneal epithelial cells, including keratosis and exfoliation, which are related to acute axonal injury during corneal nerve damage [4] and lead to symptoms such as pain and photophobia [5]. In vivo confocal microscopy (IVCM) examinations of DED patients have also revealed loss of parallelization and disordered arrangement in the corneal subepithelial nerve [5, 6].

Vitamin B1 (thiamine) given perineurally exerts analgesic effects through axonal flow by enhancing the synthesis of acetylcholine in dorsal horn inhibitory neurons [7, 8]. In addition, vitamin B12 can repair peripheral nerves by inducing the proliferation of Schwann cells and regeneration of myelinated nerve fibers. Cobalamin is the endogenous form of vitamin B12 and is superior to other forms of vitamin B12, such as cyanocobalamin and cobalamin adenosine, for the treatment of peripheral neuropathy [9, 10]. However, the effects of oral vitamin B1 and cobalamin on DED have not been investigated. Some studies have shown that local application of vitamin B12 is effective for corneal recovery and reduces corneal neuropathic pain [11]. Nevertheless, it may be insufficient to evaluate the efficacy of DED treatment using only objective clinical indicators, as patient satisfaction is also extremely important in the treatment process [12, 13]. Therefore, the purpose of this study was to investigate the effects of oral vitamin B1 and cobalamin on signs and symptoms of dry eye and treatment satisfaction in patients with DED in order to help tailor the treatment strategy to patient needs.

## 2. Methods

The study was approved by the Human Research and Ethics Committee of Peking University Third Hospital (No. M2018149), adhered to the tenets of the Declaration of Helsinki, and was performed at Peking University Third Hospital Eye Center from October 2018 to March 2019. Written informed consent was obtained from each patient before enrollment in the study.

### 2.1. Participants

A randomized controlled trial was conducted at Peking University Third Hospital. DED was diagnosed according to the diagnostic criteria established by the Tear Film and Ocular Surface Society (TFOS) Dry Eye WorkShop II (DEWS II) [14]. Inclusion criteria included positive symptoms (Dry Eye Questionnaire-5 > 5 or ocular surface disease index (OSDI) > 13) and at least one of the following signs: noninvasive breakup time (BUT) < 10 seconds; osmolarity > 308 mOsm/L and interocular difference > 8 mOsm/L; and corneal staining > 5 spots and conjunctival staining > 9 spots. Patients who had other corneal diseases, glaucoma, macular degeneration, optic neuropathy, uveitis, diabetic retinopathy, or complex systemic disease or were incapable of carrying out study-related visits were excluded.

### 2.2. Experimental Design

**Figure 1:**
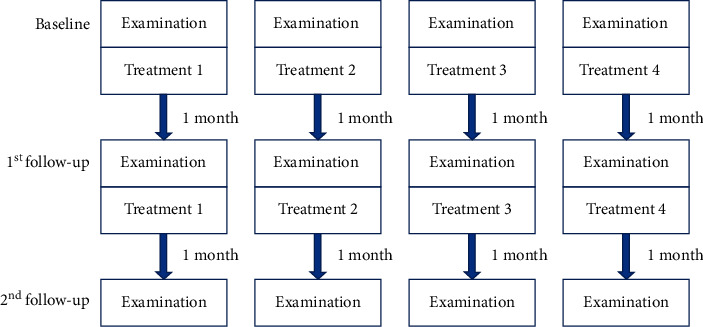
Treatment 1 (group 1): only artificial tears; treatment 2 (group 2): corticosteroids and artificial tears; treatment 3 (group 3): oral vitamin B1, mecobalamin, and artificial tears; treatment 4 (group 4): oral vitamin B1, mecobalamin, corticosteroids, and artificial tears.

DED patients with corneal nerve layer injury were divided into the Langerhans group and non-Langerhans group based on the presence of Langerhans cells in the corneal nerve layer on IVCM. Patients in the former group were administered with corticosteroid eye drops (Flumetholon, Saten, Japan) and randomly assigned to receive oral vitamin B1 (vitamin B1 tablets, Shanxi Hengruida Pharmaceutical Co., China), mecobalamin (mecobalamin tablets, Weicai (China) Pharmaceutical Co., China), and/or artificial tears (Hycosan, URSAPHARM, Germany, and Systane Ultra Lubricant Eye Drops, Alcon Laboratories, USA). Patients in the latter group were randomly assigned to receive oral vitamin B1, mecobalamin, and/or artificial tears (Hycosan, URSAPHARM, Germany, and Systane Ultra Lubricant Eye Drops, Alcon Laboratories, USA) or artificial tears alone as a control. Thus, the patients were ultimately divided into 4 groups: group 1, only artificial tears; group 2, corticosteroid eye drops and artificial tears; group 3, oral vitamin B1, mecobalamin, and artificial tears; and group 4, oral vitamin B1, mecobalamin, corticosteroid eye drops, and artificial tears. Each patient underwent continuous treatment for 2 months and was subjected to clinical assessment before treatment (baseline) and at 1 and 2 months after treatment. The treatments and follow-up protocols for all groups are shown in Figure 1.

### 2.3. Evaluation Index

At every follow-up, each participant completed a questionnaire regarding subjective symptoms and satisfaction with treatment [15]. Slit lamp examination and other objective and auxiliary screenings were conducted to evaluate signs of DED. Each examination was carried out in the same examination room under the same conditions by a single doctor.

A Placido ring-based, noncontact corneal topographer (Keratograph 5 M; OCULUS, Wetzlar, Germany) was used to evaluate the tear film [16], including the tear meniscus height (TMH), tear film breakup time first (TBUTF), and tear film breakup time average (TBUTA). In order to ensure a dark background in corneal topography, automatic red-ring illumination was used. An illuminated ring pattern was projected onto the cornea using the corneal topographer, which features a 22-ring Placido disk on which four infrared diodes arranged in two parallel rows are fixed horizontally. To avoid errors, the examination was repeated three times. During each examination, patients were asked to keep their eyes open for as long as possible after 3 to 4 blinks. From this point on, the examiner began to observe the corneal reflex image. When tear film breakup occurred or became unstable, the reflection image became irregular, and the tear film breakup time was obtained. The first value was the TBUTF, and the mean of three values was the TBUTA. The distance between the light band at the junction of the corneal conjunctival surface and the lower eyelid margin was the TMH.

IVCM was used to observe the microstructure of the cornea. All patients were examined with a digital corneal confocal laser-scanning microscope (HRT II RCM Heidelberg Engineering Inc., Heidelberg, Germany, Rostock Cornea Module) equipped with the built-in software Heidelberg Eye Explorer version 1.5.10.0. Two-dimensional images with a definition of 384 ∗ 384 pixels over an area of 400 *μ*m ∗ 400 *μ*m, lateral spatial resolution of 0.5 *μ*m, and depth resolution of 1-2 *μ*m were captured. For each eye, 30–40 images were acquired from the corneal epithelium to the endothelium. Five images of good quality were chosen to quantify corneal nerves using a pretrained deep learning model [17], and the average corneal nerve fiber density (CNFD) was recorded.

All patients were asked to complete two questionnaires to evaluate their symptoms and satisfaction with treatment before and at 1 month and 2 months after treatment. The patients responded to the questions using a 10-point scale. The first questionnaire comprised 10 questions (questions 1–10) related to 10 DED symptoms, including dryness, foreign body sensation, pain, burning, watering, asthenopia, blurred vision, itching, increased secretions, and photophobia. The responses ranged from “have no feelings (score 0)” to “very serious (score 10).” The second questionnaire was related to satisfaction with treatment and included 4 questions regarding how the patients felt about their condition before and after treatment (responses ranged from “very bad (score 0)” to “very good (score 10)”), the ease of following doctors' advice (from “very difficult (score 0)” to “very easy (score 10)”), and improvement after treatment (from “none (score 0)” to “significantly improved (score 10)”).

The questionnaire scores, satisfaction scores, TMH, TBUTF, TBUTA, and CNFD of each group at each time point were compared within the individual groups and between groups to evaluate the therapeutic effects of the different treatments.

### 2.4. Statistical Analysis

SPSS software version 22 (SPSS Inc., Chicago, IL, USA) was used for statistical analysis. The Kolmogorov-Smirnov test was used to check the normality of the data distribution. Descriptive data are presented as the mean and standard deviation (SD). The mixed linear model was used to compare differences before and after treatment. To analyze repeated measurements, the patient was selected as the subject, and the time point was set as the repeated factor. In the model, the covariance type was chosen based on the covariance matrix from our preliminary analysis. *P* values less than 0.05 were considered statistically significant.

## 3. Results

### 3.1. Patient Characteristics

A total of 152 eyes from 76 individuals (age, 55.25 ± 18.16 years; 56 females) were included and divided into 4 groups according to treatment. Significant age and sex differences were not found among the groups. The demographic characteristics of the patients are shown in Table 1.

### 3.2. Comparison of Pre- and Posttreatment Signs, Symptoms, and Treatment Satisfaction Scores in Each Group

**Figure 2:**
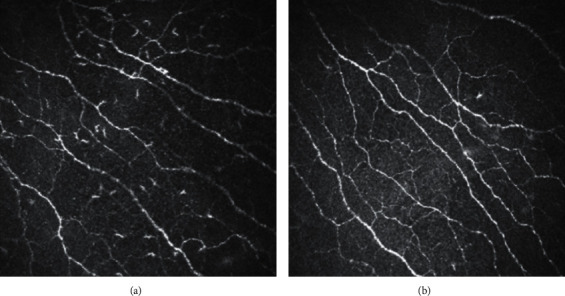
These two images are from the same corneal nerve layer of subepithelial corneal epithelium of the IVCM in the same patient in group 4: (a) before treatment; (b) 1 month after treatment.

As shown in Table 2, in group 3, dryness, foreign body sensation, burning, and TBUTF differed significantly between 1 and 2 months after treatment, and satisfaction scores differed significantly before and after treatment (*P* < 0.05). In group 3, there were also significant differences in dryness, foreign body sensation, and photophobia and TBUTA between baseline and 2 months after treatment (*P* < 0.05). In group 4, pain, blurred vision, and the total symptom score were significantly different between baseline and 1 month after treatment (*P* < 0.05), and asthenopia differed significantly between 1 and 2 months after treatment (3.97 ± 3.34, 5.23 ± 3.19, *P* < 0.05). Interestingly, in both groups 3 and 4, CNFD increased at 1 and 2 months after treatment (*P* < 0.05). However, in group 4, the Langerhans cells in the corneal nerve layer were obviously reduced at 1 month after treatment, as shown in Figure 2. Finally, in group 1, significant differences were observed in dryness between baseline and 1 month after treatment and in blurred vision between 1 and 2 months after treatment and between baseline and 2 months after treatment (*P* < 0.05).

### 3.3. Comparison of Pre- and Posttreatment Signs, Symptoms, and Treatment Satisfaction Scores among Groups

As shown in Table 3, the changes in dryness between baseline and 1 and 2 months after treatment differed significantly between groups 1 and 4 (*P* < 0.05), and the changes in dryness between 1 and 2 months after treatment differed significantly between groups 2/4 and 3 (*P* < 0.05). The changes in foreign body sensation between 1 and 2 months after treatment differed significantly between groups 2/3 and 4 (*P* < 0.05), and the changes in photophobia between 1 and 2 months after treatment differed significantly between groups 2/3 and 4 (*P* < 0.05). The changes in the total symptom score between baseline and 1 and 2 months after treatment differed significantly between groups 1/2 and 4 (*P* < 0.05). In addition, the change in CNFD between baseline and 1 and 2 months after treatment differed significantly between groups 2 and 3/4 (*P* < 0.05), while the change between 1 and 2 months after treatment differed significantly between groups 1/2 and 3 (*P* < 0.05).

### 3.4. Correlations between Pre- and Posttreatment Signs/Symptoms/Treatment Satisfaction Scores and Sex/Age

The correlations between pre- and posttreatment signs/symptoms/treatment satisfaction scores and sex/age are shown in Table 4. In group 3, dryness and photophobia improved to a significantly greater extent in men, while the improvements in TBUTF and itching were significantly greater in women. TBUTF, TBUTA, burning, itching, and the total symptom score improved to a significantly greater extent in the older age group compared with the younger age group.

## 4. Discussion

DED is a multifactorial disease with various symptoms and signs [1]. Many treatments for dry eye improve only some symptoms/signs, while other symptoms, particularly pain and photophobia, are neglected. Therefore, there is a need for new convenient treatment measures. To provide new ideas for diagnosis and treatment, this study evaluated whether oral vitamin B1 and mecobalamin can improve the signs/symptoms/satisfaction of patients with DED.

In this study, the scores of four dry eye symptoms (dryness, foreign body sensation, burning, and photophobia) and TBUTF improved significantly at 2 months after treatment with oral vitamin B1, mecobalamin, and artificial tears (group 3). When corticosteroid eye drops were added to this treatment (group 4), pain, blurred vision, and total symptom scores were significantly improved at 1 month after treatment. We also observed an obvious decrease in Langerhans cells in the corneal nerve layer at 1 month after treatment. In addition, the CNFD proved obviously improved in both groups 3 and 4 at 1 and 2 months after treatment. These observations suggest that oral vitamin B1 and mecobalamin can help nourish and repair the corneal nerve layer to some extent, thereby alleviating burning and photophobia.

The cornea possesses approximately a hundred times more nociceptors than the dermis. These nociceptors are located slightly below the lacrimal layer, between the epithelial cells, and are unmyelinated, leading to extreme sensitivity in nociceptive sensation [18, 19]. The severity of dry eye may depend on the degree of changes in the structure and function of the subcorneal nerve [20]; greater severity of dry eye is associated with a larger degree of changes in nerve branches and curvature on IVCM [21]. Ocular surface neuropathy can induce or aggravate dry eye, and long-term serious dry eye can produce changes in the structure and function of ocular surface nerves, resulting in a vicious circle [5, 21]. Corneal nerve injury can lead to acute axonal injury, which can reduce the threshold potential of ion channels in corneal nerve endings due to the release of inflammatory mediators such as substance *P*, tumor necrosis factor-*α*, and interleukin-1 [4]. The simultaneous release of nerve growth factor during this process leads to gene expression and upregulation, resulting in an excessive corneal nociceptor response [4], which might account for symptoms of pain, burning, and photophobia. By contrast, repeated or severe axonal injury can shift the corneal nociceptor's functional state to nerve regeneration rather than signal transduction [18, 22], temporarily alleviating symptoms. During regeneration, the damaged nerves can form nerve bundles and microneuromas, which may also cause spontaneous pain, burning, and photophobia [18].

Previous studies have shown that the analgesic effect of vitamin B1 occurs via indirect enhancement of axonal flow [7, 8, 23]. We believe that, in the corneal nerve, this effect of vitamin B1 can lead to relief of pain. Cobalamin, an endogenous form of vitamin B12, is present in the blood and medullary fluid. Compared with other forms of vitamin B12, cobalamin has superior effects on neuronal transmission. Cobalamin can promote nucleic acid, protein, and lipid metabolism through methyl conversion reactions [10]. As a coenzyme, cobalamin participates in the synthesis of thymine from deoxynucleoside, promotes the metabolism of nucleic acid protein through methyl transformation reactions, serves as a cofactor in the conversion of homocysteine to methionine, and promotes the formation of the axonal myelin sheath and axonal transport, thus repairing damaged nerve tissue [9]. Vitamin B12 is widely used in the treatment of pain, including diabetic neuropathy, herpes, and surgery-related pain. Some researchers have reported that patients with vitamin B12 deficiency and ocular neuropathic pain respond effectively to vitamin B12 treatment and are able to discontinue all topical medications within a few weeks of treatment [24]. By contrast, a period of two months was required to observe effectiveness in the treatment group in the present study. This discrepancy may be related to the mode of administration and interactions with other drugs. Some in vivo studies have shown that the analgesic effect of vitamin B12 is due to the activation of opioid receptors or increased serotonin levels in different regions of the brain, which enhance the inhibitory effect of afferent noxious neurons in the spinal cord and, in turn, lead to weakening of the thalamic neuronal response to noxious stimuli. In addition, vitamin B12 eye drops have been reported to alleviate dry eye symptoms by reducing oxidative stress and inflammation [25]. However, there are no reliable studies of its pharmacokinetics, and hence we cannot conclude whether the effectiveness of local instillation differs from that of oral administration. Vitamin B12 has also been reported to improve corneal reinnervation and reepithelization after injury [26], and the changes in CNFD observed in the present study partially confirm these findings.

The significant differences in satisfaction scores between baseline and 1 and 2 months after treatment in group 3 are consistent with previous reports and are not unexpected because photophobia, burning, and pain are subjective symptoms and have a greater impact on satisfaction scores [12, 27]. Corticosteroid supplementation has also been shown to be helpful in reducing ocular surface inflammation, as corticosteroids inhibit proinflammatory cytokines [28]. Corticosteroids exert a powerful anti-inflammatory effect by acting on subbasal dendritic cells in the corneal nerve layer and activated Langerhans cells, both of which play roles in inflammation [29, 30]. This explains the beneficial effects of corticosteroids on group 4, which included significant improvements in pain, blurred vision, and total symptom scores and were similar to the results of earlier studies [29]. The significant reduction in Langerhans cells in the corneal nerve layer in group 4 at 1 month after treatment partially supports this conjecture. However, similar changes were not observed in group 2, in which both corticosteroid eye drops and artificial tears were given, suggesting a synergistic effect of corticosteroids, vitamin B1, and mecobalamin. However, withdrawal of the corticosteroid after 1 month may have led to rebound of some symptoms, which has rarely been reported in previous studies.

Unexpectedly, demographic differences in the effectiveness of the treatments for DED were observed: oral vitamin B1 and mecobalamin were more effective for men than women, particularly for dryness and photophobia. We speculate that this may be related to the decline in hormone levels in older women, but additional experiments are needed to confirm this possibility. These findings could be used to guide treatment in the future.

In summary, our study showed that oral vitamin B1 and mecobalamin can relieve some DED symptoms such as dryness, foreign body sensation, and photophobia; stabilize the tear film; and improve patient satisfaction with regard to treatment. We speculate that neurotrophism may be one of the mechanisms underlying these effects. However, at least 2 months of treatment were required for these benefits to be evident, which demands patient compliance. Corticosteroid eye drops can help relieve the overall symptoms of dry eyes, but any withdrawal may cause rebound.

Our study provides new therapeutic options for the treatment of DED as well as guidance regarding the duration and combined use of different DED treatments. A limitation of this study is the relatively small sample size. In addition, this was a trial treatment, and we did not check serum vitamin B1 and vitamin B12 levels in the DED patients. We do not know whether there is synergy or interaction between vitamin B1 and mecobalamin, or do we know which is more effective in the treatment of DED. Answering these questions will be the focus of our subsequent research. As noted previously [11, 30], continuous patient education and counseling should be combined with pharmaceutical treatment for the management of complex diseases such as DED.

## Figures and Tables

**Table 1:** Basic information.

	Group 1	Group 2	Group 3	Group 4	*P*
Sex (male %)	20	30	25	31.25	ns
Age (years, mean ± SD)	57.35 ± 16.37	51.90 ± 18.57	53.85 ± 17.93	58.56 ± 19.40	ns
Number	40	40	40	32	

ns: no statistical significance.

**Table 2:** Comparison of pre- and posttreatment in each group.

		Group 1			Group 2			Group 3			Group 4		
		*B*	1	2	*B*	1	2	*B*	1	2	*B*	1	2
Signs (mean ± SD)	TMH (mm)	0.18 ± 0.05	0.20 ± 0.07	0.21 ± 0.07	0.16 ± 0.06	0.18 ± 0.07	0.17 ± 0.07	0.18 ± 0.05	0.17 ± 0.06	0.18 ± 0.07	0.17 ± 0.05	0.19 ± 0.06	0.18 ± 0.07
	TBUTF (s)	5.19 ± 2.65	5.23 ± 2.20	5.85 ± 2.60	4.70 ± 1.80	4.87 ± 2.37	6.12 ± 3.81	4.21 ± 1.21	4.89 ± 1.80	5.31 ± 2.07^#^	3.98 ± 1.80	4.55 ± 1.62	5.10 ± 2.74
	TBUTA (s)	6.60 ± 2.85	7.06 ± 2.15	7.55 ± 3.28	5.93 ± 1.79	6.15 ± 2.15	7.55 ± 3.28^∂#^	5.70 ± 1.71	6.46 ± 1.48^*∗*^	6.44 ± 2.33	5.09 ± 1.97	5.95 ± 1.95	6.71 ± 2.78
	CNFD (mm/mm^2^)	14.14 ± 2.84	16.56 ± 1.76	17.51 ± 1.05	13.47 ± 1.14	13.33 ± 1.93	14.36 ± 1.61	6.54 ± 1.85	9.71 ± 2.49^*∗*^	13.93 ± 2.59^#^	7.11 ± 2.48	10.55 ± 2.12^*∗*^	15.98 ± 1.72^#^

Symptoms (mean ± SD, scores)	Dryness	5.13 ± 3.41	6.03 ± 3.03^*∗*^	5.83 ± 3.61	6.15 ± 3.14	5.58 ± 2.92	5.70 ± 2.79	6.15 ± 3.07	6.43 ± 3.54	4.69 ± 3.30^∂#^	5.88 ± 3.13	4.47 ± 3.32	4.64 ± 3.03
	Foreign body sensation	3.70 ± 3.70	4.88 ± 3.47	4.88 ± 3.40	3.38 ± 3.61	3.30 ± 3.07	3.98 ± 3.36	5.38 ± 3.64	5.30 ± 3.43	3.42 ± 3.41^∂#^	4.66 ± 3.76	3.47 ± 3.79	3.59 ± 3.57
	Pain	1.95 ± 3.00	1.50 ± 2.61	2.00 ± 2.88	2.65 ± 3.18	2.75 ± 2.97	2.83 ± 3.03	3.63 ± 3.18	2.80 ± 3.30	2.04 ± 2.86	4.03 ± 3.61	2.53 ± 3.29^*∗*^	3.82 ± 3.57
	Burning	1.15 ± 1.86	1.65 ± 2.77	1.45 ± 2.73	2.25 ± 3.18	2.40 ± 2.99	2.93 ± 2.74	2.10 ± 3.18	2.33 ± 3.34	0.96 ± 1.97^#^	2.47 ± 3.65	1.44 ± 2.33	1.27 ± 2.90
	Watering	2.10 ± 2.92	2.50 ± 3.10	2.48 ± 3.03	2.30 ± 3.04	2.28 ± 3.04	2.33 ± 2.78	4.05 ± 3.59	3.33 ± 3.87	3.73 ± 3.54	1.94 ± 3.15	2.16 ± 3.33	2.00 ± 3.27
	Asthenopia	4.50 ± 3.80	5.30 ± 3.07	5.10 ± 3.39	5.75 ± 3.46	6.23 ± 2.95	5.75 ± 3.20	5.65 ± 3.22	6.00 ± 3.22	4.96 ± 3.37	4.31 ± 3.76	3.97 ± 3.34	5.23 ± 3.19^∂^
	Blurred vision	3.55 ± 3.56	2.20 ± 3.12	3.60 ± 3.51^#^	3.10 ± 3.62	3.45 ± 3.04	3.20 ± 3.43	3.13 ± 3.49	2.85 ± 3.42	3.81 ± 3.37	3.97 ± 4.04	2.81 ± 3.41^*∗*^	3.41 ± 3.59
	Itching	2.85 ± 2.89	2.60 ± 2.47	3.15 ± 2.98	2.15 ± 3.45	2.25 ± 3.10	3.48 ± 3.23	2.98 ± 3.33	3.23 ± 3.18	2.58 ± 3.20	2.59 ± 3.26	1.88 ± 2.65	1.91 ± 2.86
	Increased secretions	1.25 ± 2.81	1.05 ± 2.63	1.23 ± 1.82	1.35 ± 2.72	2.48 ± 3.45	2.20 ± 2.59	2.43 ± 3.61	2.48 ± 3.22	3.27 ± 2.99	3.66 ± 3.53	3.28 ± 3.74	2.73 ± 3.38
	Photophobia	3.40 ± 3.82	3.95 ± 3.74	3.33 ± 3.59	4.80 ± 3.69	4.90 ± 3.64	4.03 ± 3.29	5.00 ± 3.82	4.63 ± 3.93	3.54 ± 3.39^#^	4.84 ± 3.90	3.75 ± 3.67	3.68 ± 3.71
	Total	29.58 ± 17.97	31.33 ± 14.67	32.95 ± 19.70	33.88 ± 12.88	35.60 ± 17.47	36.40 ± 15.38	40.48 ± 21.45	39.35 ± 20.73	32.73 ± 23.33	38.34 ± 23.67	29.75 ± 23.07^*∗*^	32.27 ± 25.31

Satisfaction (mean ± SD, scores)	Before	4.00 ± 2.63	3.60 ± 2.99	3.82 ± 2.60	4.50 ± 2.40	4.22 ± 2.50	4.20 ± 2.56	4.41 ± 2.97	3.40 ± 2.19	2.82 ± 1.30	3.29 ± 2.59	3.60 ± 2.17	3.50 ± 3.13
	After		5.21 ± 2.62	5.72 ± 2.21		5.67 ± 2.32	6.05 ± 2.14		5.87 ± 2.62	6.27 ± 2.10		4.67 ± 1.67	5.31 ± 3.03
	After-before		1.43 ± 2.39	1.89 ± 1.97		1.44 ± 1.92	1.85 ± 1.39		2.47 ± 2.65	3.45 ± 2.24^∂^		0.50 ± 1.77	2.00 ± 2.18
	Following advice		9.07 ± 2.33	8.94 ± 2.18		8.83 ± 1.44	8.50 ± 1.55		9.53 ± 1.28	9.55 ± 0.80		9.86 ± 0.36	9.29 ± 1.20
	Improvement		6.00 ± 3.11	5.56 ± 3.16		6.28 ± 3.19	6.80 ± 2.89		6.33 ± 2.77	7.36 ± 2.32		6.57 ± 2.34	5.86 ± 3.90

B: baseline; 1: 1 month; 2: 2 months; TMH: tear meniscus height; TBUTF: tear film breakup time first; TBUTA: tear film breakup time average; CNFD: corneal nerve fiber density. Satisfaction: before: pretreatment satisfaction with ocular surface; after: posttreatment satisfaction with ocular surface; following advice: compliance with the doctor's advice; improvement: satisfaction with therapeutic effects. *P* values of less than 0.05 were considered statistically significant and are expressed as ^*∗*^1 month versus baseline, ^#^2 months versus baseline, and ^∂^2 months versus 1 month in the same group.

**Table 3:** Comparison of pre- and posttreatment among the groups.

		1 month-baseline					2 months–-baseline					2 months-1 month				
		G1	G2	G3	G4	*P*	G1	G2	G3	G4	*P*	G1	G2	G3	G4	*P*
Signs (mean ± SD)	TMH (mm)	0.02 ± 0.06	0.02 ± 0.06	−0.01 ± 0.06	0.02 ± 0.07		0.03 ± 0.07	0.01 ± 0.06	0.01 ± 0.05	0.00 ± 0.08		0.01 ± 0.06	−0.01 ± 0.06	0.01 ± 0.08	−0.01 ± 0.06	
	TBUTF (s)	0.04 ± 3.49	0.17 ± 2.35	0.69 ± 2.04	0.59 ± 1.76		0.66 ± 3.79	1.42 ± 4.26	1.15 ± 1.57	0.79 ± 2.74		0.62 ± 3.39	1.25 ± 3.42	0.48 ± 1.86	0.66 ± 2.54	
	TBUTA (s)	0.46 ± 2.87	0.22 ± 2.50	0.74 ± 1.97	0.43 ± 1.77		0.62 ± 3.38	1.62 ± 3.82	0.97 ± 2.43	1.37 ± 2.67		0.16 ± 2.68	1.40 ± 2.96	0.02 ± 1.51	0.82 ± 2.71	
	CNFD (mm/mm^2^)	2.42 ± 1.75	−0.13 ± 1.66	3.16 ± 1.94^ε^	3.44 ± 3.55^∂^	0.021	3.37 ± 2.60	0.89 ± 1.07	7.39 ± 3.57^ε^	9.32 ± 3.00^∂^	0.001	0.95 ± 1.43	1.03 ± 1.53	4.22 ± 2.83^*∗ε*^	4.93 ± 3.21	0.002

Symptoms (mean ± SD, scores)	Dryness	0.90 ± 2.11	−0.58 ± 2.52	0.28 ± 3.03	−1.41 ± 3.64^#^	0.005	0.70 ± 2.39	−0.45 ± 2.96	−0.81 ± 2.37	−1.18 ± 2.65^#^	0.028	−0.20 ± 2.54	0.13 ± 2.48	−1.81 ± 2.47^ε^	0.23 ± 2.99^∀^	0.015
	Foreign body sensation	1.18 ± 3.21	−0.08 ± 3.27	−0.08 ± 3.11	−1.19 ± 3.11^#^	0.021	1.18 ± 3.38	0.60 ± 3.16	−1.31 ± 3.65	−1.50 ± 5.18^#^	0.011	0.00 ± 2.65	0.68 ± 3.12	−1.88 ± 3.14^ε^	−0.91 ± 4.25	0.013
	Pain	−0.45 ± 2.16	0.10 ± 3.57	−0.83 ± 3.17	−1.50 ± 2.51		0.05 ± 3.43	0.18 ± 3.46	−1.38 ± 3.09	−0.91 ± 3.71		0.50 ± 3.21	0.08 ± 3.44	−0.46 ± 2.45	0.91 ± 3.50	
	Burning	0.50 ± 3.00	0.15 ± 3.33	0.23 ± 2.28	−1.03 ± 3.03		0.30 ± 2.57	0.68 ± 3.12	0.12 ± 1.93	−0.55 ± 2.44		−0.20 ± 3.39	0.53 ± 2.97	−0.46 ± 2.25	−0.09 ± 1.23	
	Watering	0.40 ± 2.91	−0.03 ± 2.11	−0.73 ± 2.41	0.22 ± 1.56		0.38 ± 2.25	0.03 ± 2.79	0.00 ± 3.01	0.55 ± 2.22		−0.03 ± 2.09	0.05 ± 3.39	0.23 ± 3.02	0.41 ± 1.22	
	Asthenopia	0.80 ± 3.67	0.48 ± 2.37	0.35 ± 2.66	−0.34 ± 2.72		0.60 ± 3.88	0.00 ± 2.31	−0.31 ± 2.77	0.77 ± 2.41		−0.20 ± 2.15	−0.48 ± 2.54	−1.00 ± 3.20	1.41 ± 2.13	0.009
	Blurred vision	−1.35 ± 3.08	0.35 ± 4.33	−0.28 ± 3.49	−1.16 ± 2.07		0.05 ± 3.59	0.10 ± 3.89	0.46 ± 1.70	−1.32 ± 2.83		1.40 ± 3.24	−0.25 ± 2.85	1.50 ± 3.48	−0.23 ± 1.85	0.020
	Itching	−0.25 ± 2.70	0.10 ± 3.95	0.25 ± 3.09	−0.72 ± 2.56		0.30 ± 2.52	1.33 ± 4.37	−0.46 ± 3.11	−0.82 ± 3.06		0.55 ± 1.91	1.23 ± 3.91	0.23 ± 3.18	0.32 ± 1.46	
	Increased secretions	−0.20 ± 1.14	1.13 ± 3.13	0.05 ± 2.77	−0.38 ± 2.55	0.045	−0.03 ± 2.12	0.85 ± 3.67	0.54 ± 3.85	−0.23 ± 1.90		0.18 ± 1.91	−0.28 ± 2.75	0.54 ± 2.92	0.09 ± 1.19	
	Photophobia	0.55 ± 2.23	0.10 ± 3.68	−0.38 ± 1.44	−1.09 ± 2.98		−0.08 ± 2.37	−0.78 ± 3.35	−1.08 ± 2.67	−0.95 ± 2.21		−0.63 ± 1.82	−0.88 ± 2.84	−0.81 ± 1.88	0.50 ± 2.94^∂∀^	
	Total	1.75 ± 13.11	1.73 ± 17.65	−1.13 ± 14.26	−8.59 ± 15.46^∂#^	0.017	3.38 ± 11.51	2.53 ± 13.08	−4.23 ± 13.60	−6.14 ± 15.06^#^	0.011	1.63 ± 11.89	0.80 ± 16.35	−3.92 ± 13.11	2.64 ± 8.94	

Satisfaction (mean ± SD, scores)	Before	−0.57 ± 3.03	−0.71 ± 2.35	−0.59 ± 4.11	0.00 ± 3.71		−0.63 ± 2.68	−0.21 ± 3.48	−0.92 ± 3.00	−0.13 ± 4.94		0.21 ± 3.13	−0.22 ± 3.84	−0.88 ± 2.50	−0.13 ± 1.73	
	After											0.46 ± 2.10	0.17 ± 3.42	0.63 ± 3.26	1.38 ± 1.51	
	Following advice											−0.14 ± 0.65	−0.28 ± 1.43	−0.38 ± 0.72	−0.50 ± 0.93	
	Improvement											−0.07 ± 2.68	0.33 ± 2.44	0.38 ± 2.93	0.25 ± 1.58	

G1: group 1; G2: group 2; G3: group 3; G4: group 4; TMH: tear meniscus height; TBUTF: tear film breakup time first; TBUTA: tear film breakup time average; CNFD: corneal nerve fiber density. Satisfaction: before: pretreatment satisfaction with ocular surface; after: posttreatment satisfaction with ocular surface; following advice: compliance with the doctor's advice; improvement: satisfaction with therapeutic effects. *P* values of less than 0.05 were considered statistically significant and are expressed as ^*∗*^groups 1 versus 3, ^#^groups 1 versus 4, ^ε^groups 2 versus 3, ^∂^groups 2 versus 4, and ^∀^groups 3 versus 4 among the different groups.

**Table 4:** The correlations between pre- and posttreatment signs/symptoms/treatment satisfaction scores and sex/age.

		G1		G2		G3		G4	
		Estimate (sex)	Estimate (age)	Estimate (sex)	Estimate (age)	Estimate (sex)	Estimate (age)	Estimate (sex)	Estimate (age)
Signs	TMH	Ns	ns	0.056	ns	ns	ns	ns	ns
	TBUTF	Ns	ns	ns	−0.029	0.719	−0.025	ns	ns
	TBUTA	ns	ns	ns	ns	ns	−0.034	ns	ns

Symptoms	Dryness	ns	ns	ns	−0.065	−1.892	ns	−2.182	ns
	Pain	ns	−0.060	ns	−0.891	ns	ns	ns	−0.083
	Burning	ns	ns	ns	ns	ns	−0.048	−2.072	ns
	Watering	ns	ns	ns	ns	ns	ns	−2.734	ns
	Asthenopia	ns	ns	ns	−0.085	ns	ns	ns	−0.054
	Blurred vision	ns	ns	1.820	ns	ns	ns	ns	0.069
	Itching	ns	ns	ns	ns	2.012	−0.091	−2.636	ns
	Photophobia	ns	ns	2.736	ns	−2.969	ns	−3.873	0.054
	Total	ns	ns	ns	ns	ns	−0.346	−26.519	ns

Satisfaction	Before	ns	ns	ns	ns	ns	0.030	−1.579	−0.113
	After	ns	ns	ns	ns	ns	−0.049	3.627	−0.055
	After-before	ns	ns	ns	ns	ns	−0.082	ns	ns
	Following advice	−2.223	ns	0.951	ns	ns	ns	−0.019	0.003
	Improvement	ns	ns	ns	ns	ns	ns	ns	−0.136

G1: group 1; G2: group 2; G3: group 3; G4: group 4; TMH: tear meniscus height; TBUTF: tear film breakup time first; TBUTA: tear film breakup time average; Satisfaction: before: pretreatment satisfaction with ocular surface; after: posttreatment satisfaction with ocular surface; following advice: compliance with the doctor's advice; improvement: satisfaction with therapeutic effects. ns: no statistical significance.

## Data Availability

The data used to support the findings of this study are available from the first author upon request.

## Abbreviations

BUT: Breakup time
DED: Dry eye disease
TBUTA: Tear film breakup time average
TBUTF: Tear film breakup time first
TMH: Tear meniscus height
IVCM: In vivo confocal microscopy
CNFD: Corneal nerve fiber density.
